# Supplementary figures and images for: Fever-like temperature bursts promote competence development via an HtrA-dependent pathway in Streptococcus pneumoniae
Source: PLoS Genet. 2023 Sep 12;19(9):e1010946. doi: 10.1371/journal.pgen.1010946 (PMC10516426; doi:10.1371/journal.pgen.1010946)

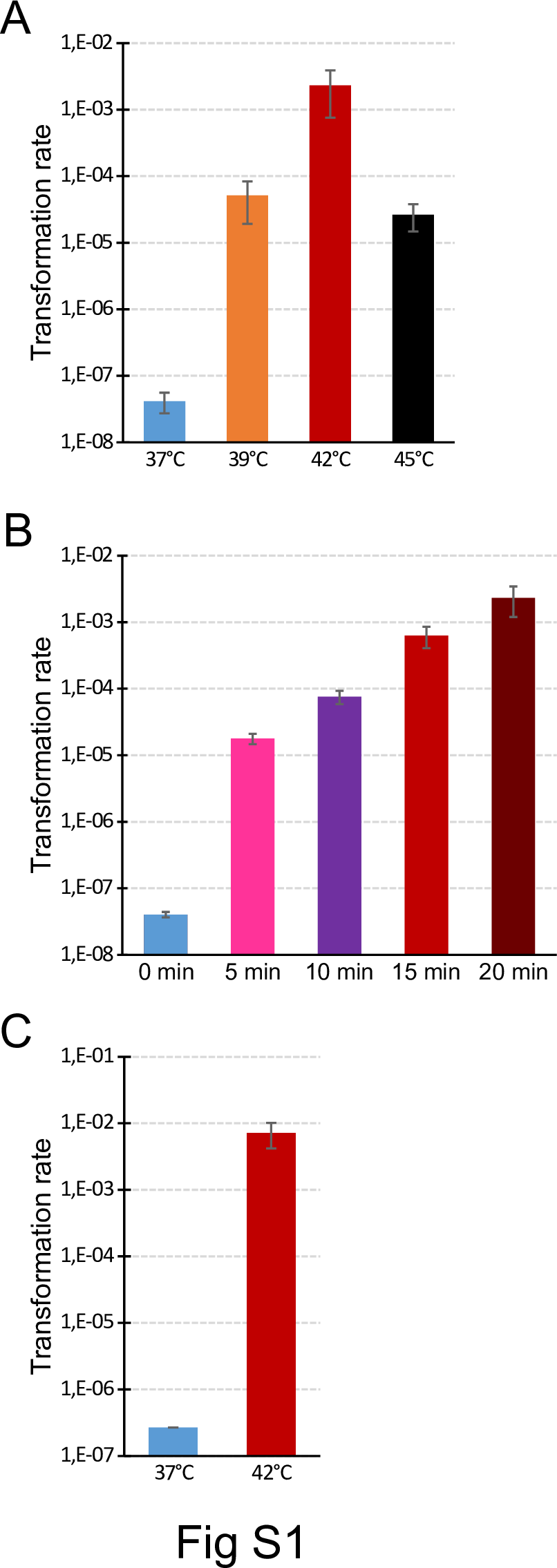

Supplement: S1 Fig — A. Comparison of transformation efficiencies in strain R825 growing in C+Y medium at 37°C after 15 minutes of exposure to 37, 39, 42 or 45°C. Saturating (100 μg mL-1) concentrations of rpsL41 PCR fragment, conferring streptomycin resistance via point mutation, was used as transforming DNA. B. As in A, but for various periods at 42°C only. C. As in A, but in strain R895 (ssb::luc) after 15 minutes at 42°C only. Data represented as Mean ± standard deviation of triplicate repeats. (TIF) [file pgen.1010946.s001.tif]

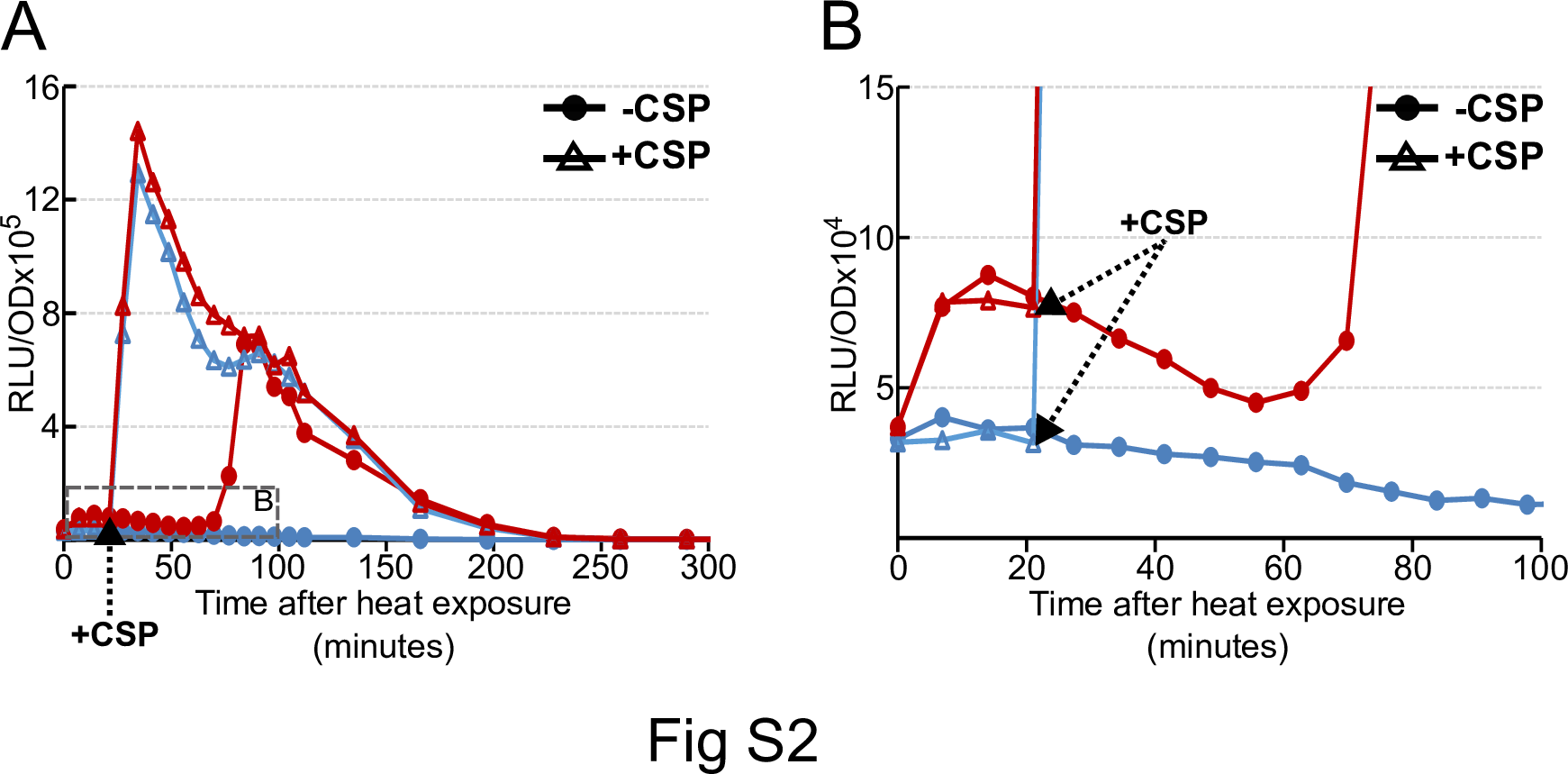

Supplement: S2 Fig — A. As in Fig 2A for strain R825 exposed for 15 minutes to 42°C (red lines) or 37°C (blue lines). CSP (100 ng mL-1) was added 20 minutes after first measurement (open triangles) or not (solid circles). For clarity, only a single data set, representative of three independent determinations made on different days, is presented. B. Enlargement of the early post-heat growth phase from panel A. Red; 15 minutes exposure to 42°C. Blue; 15 minutes exposure to 37°C. (TIF) [file pgen.1010946.s002.tif]

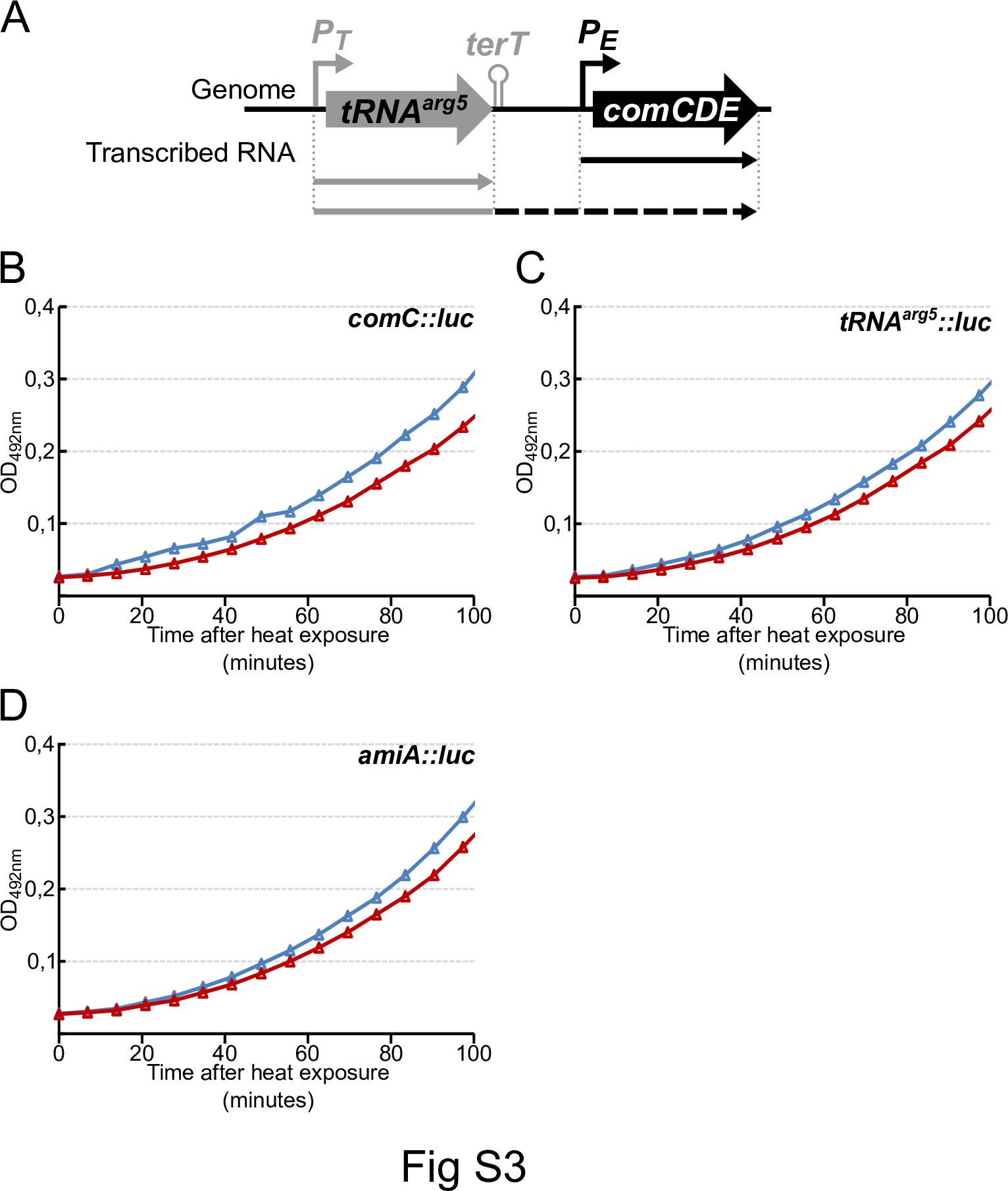

Supplement: S3 Fig — A. Schematic representation of the comCDE locus and upstream region. Promoters driving the basal level of comCDE transcription are represented by bent arrows. Straight arrows represent transcription activity; the dashed arrow represents transcription bypassing the terminator terT. B-D. Growth of pneumococcal cells in Fig 3B–3D respectively strains R1521 (comC::luc), R1694 (tRNAarg5::luc) and R4639 (amiA::luc). Red; 15 minutes exposure to 42°C. Blue; 15 minutes exposure to 37°C. (TIF) [file pgen.1010946.s003.tif]

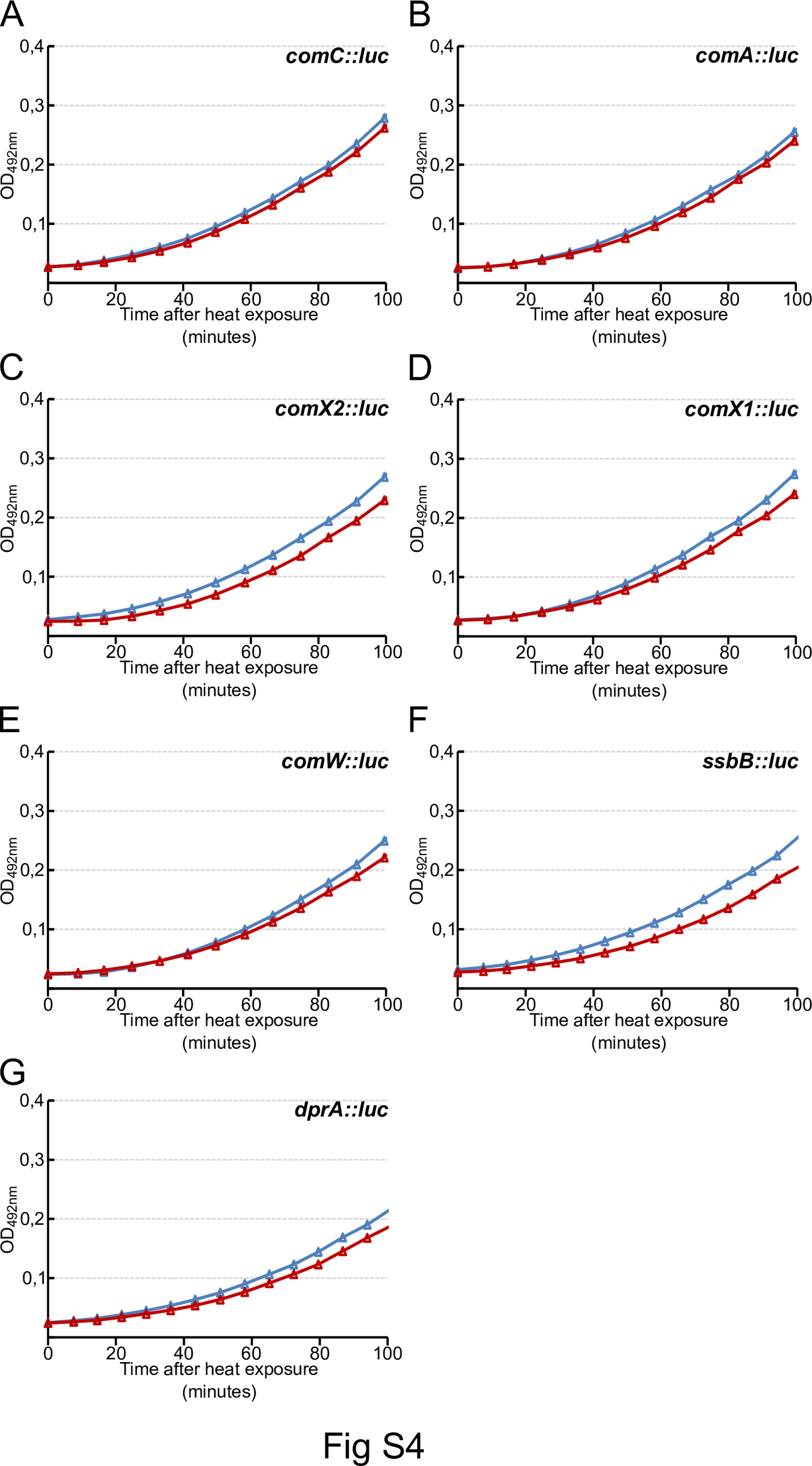

Supplement: S4 Fig — Growth of pneumococcal cells in Fig 4A–4G: A. R1521 (comC::luc); B. R1548 (comA::luc); C. R2200 (comX2::luc); D. R2218 (comX1::luc); E. R3688 (comW::luc); F. R1502 (ssbB::luc), G. R2448 (dprA::luc). Red; 15 minutes exposure to 42°C. Blue; 15 minutes exposure to 37°C. (TIF) [file pgen.1010946.s004.tif]

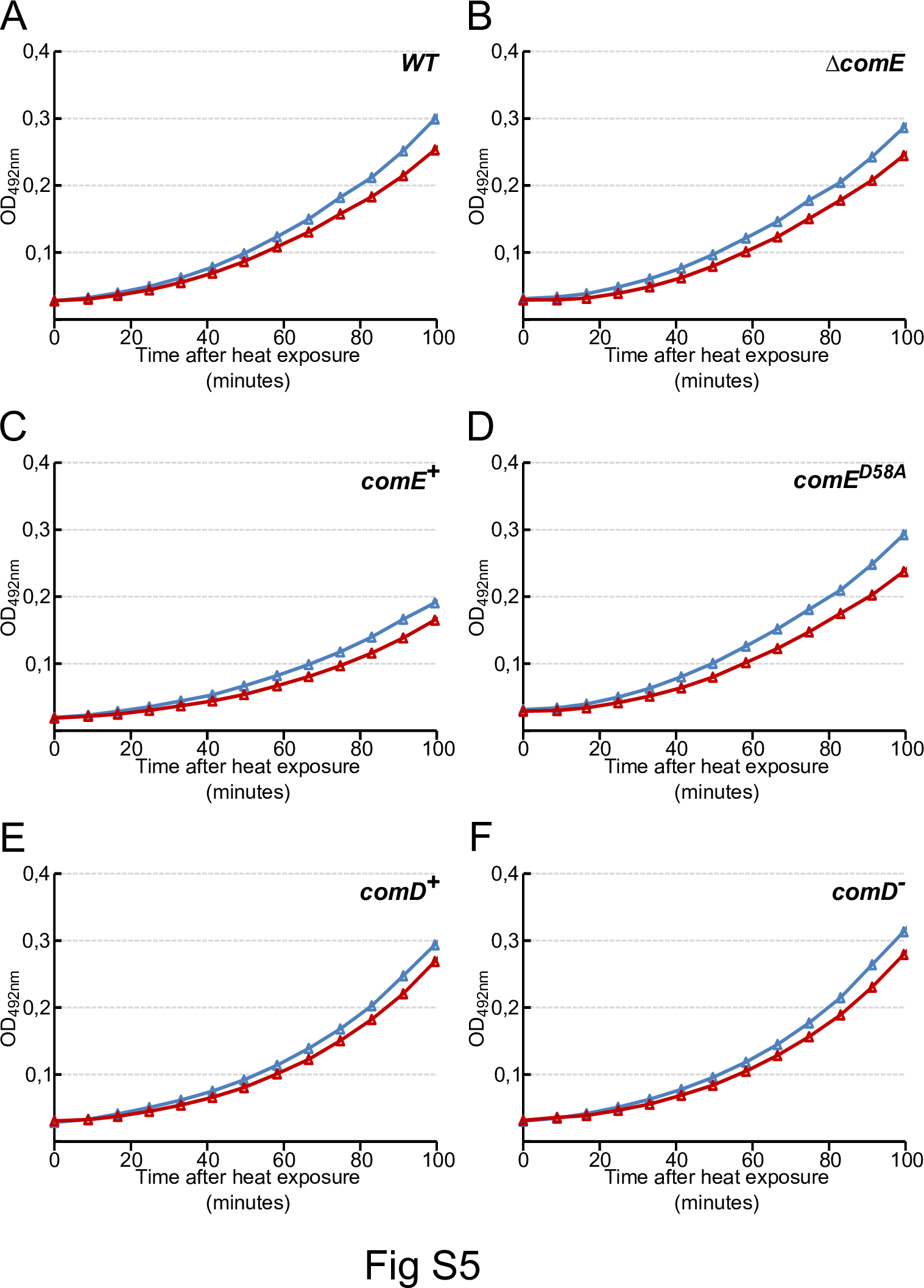

Supplement: S5 Fig — Growth of pneumococcal cells in Fig 5A–5F. A. R1521 (comC::luc); B. R1627 (comC::luc, comE-); C. 4585 (comC::luc, hexA-); D. R1798 (comC::luc, hexA-, comED58A); E. R1628 (comC::luc, comD+), F. R1648 (comC::luc, comD-). Red; 15 minutes exposure to 42°C. Blue; 15 minutes exposure to 37°C. (TIF) [file pgen.1010946.s005.tif]

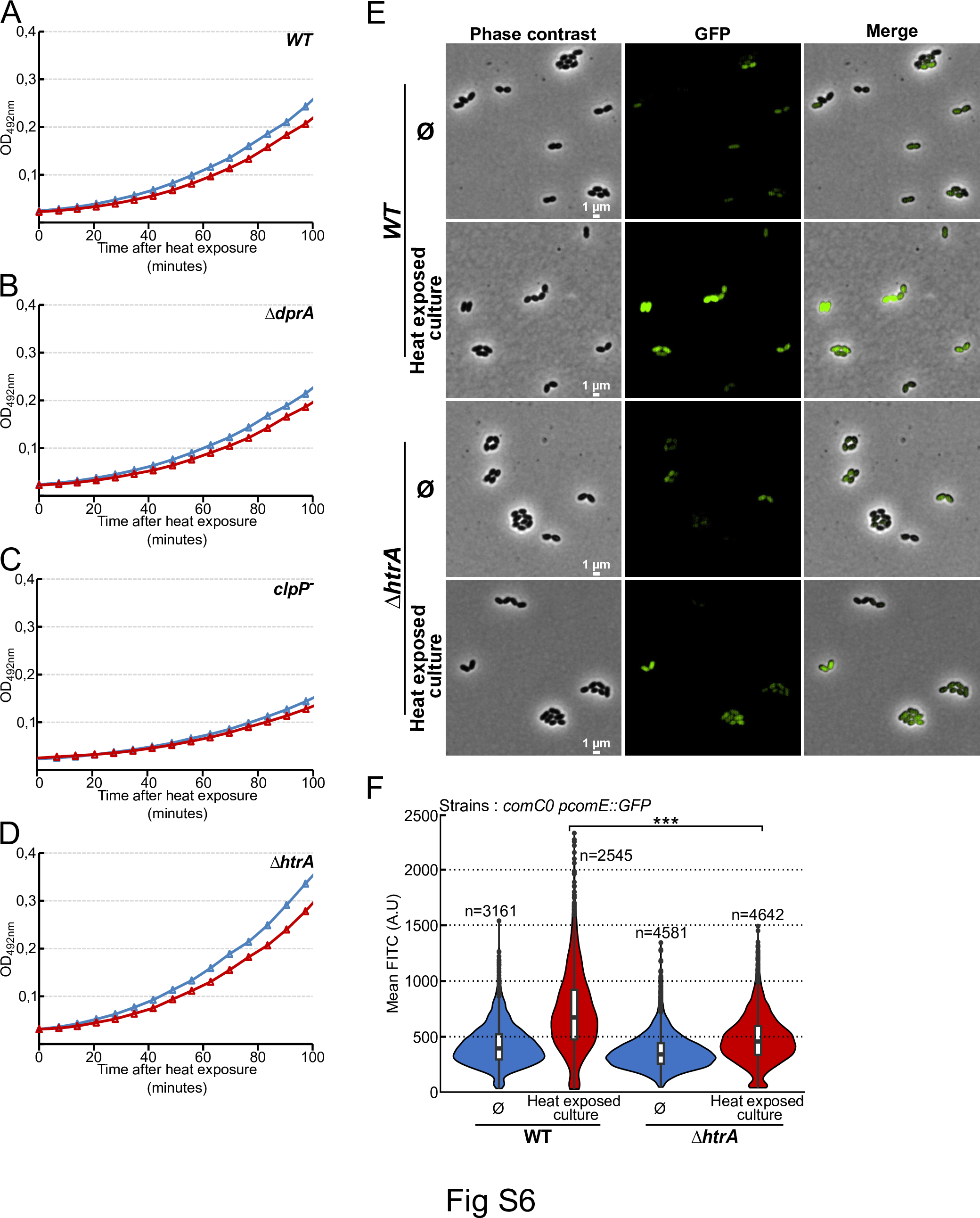

Supplement: S6 Fig — Growth of pneumococcal cells in Fig 6A–6D. A. R1521 (comC::luc); B. Strain R2017 (comC::luc, dprA-); C. R1526 (comC::luc, clpP-); D. Strain R4578 (comC::luc, htrA-). Red; 15 minutes exposure to 42°C. Blue; 15 minutes exposure to 37°C. E. Fluorescence in cells of strains R4254 and R5129 (htrA-) carrying the gfp gene under the control of a ComE-dependent promoter. F. Violin plots representing mean GFP fluorescence intensity in non-HS (blue) and HS (red) cells. Boxes extend from the 25th percentile to the 75th percentile, with the horizontal line at the median. Dots represent outliers. n = number of cells analysed for strains R4254 and R5129 in the different conditions. *** = p-value < 0.001. (TIF) [file pgen.1010946.s006.tif]

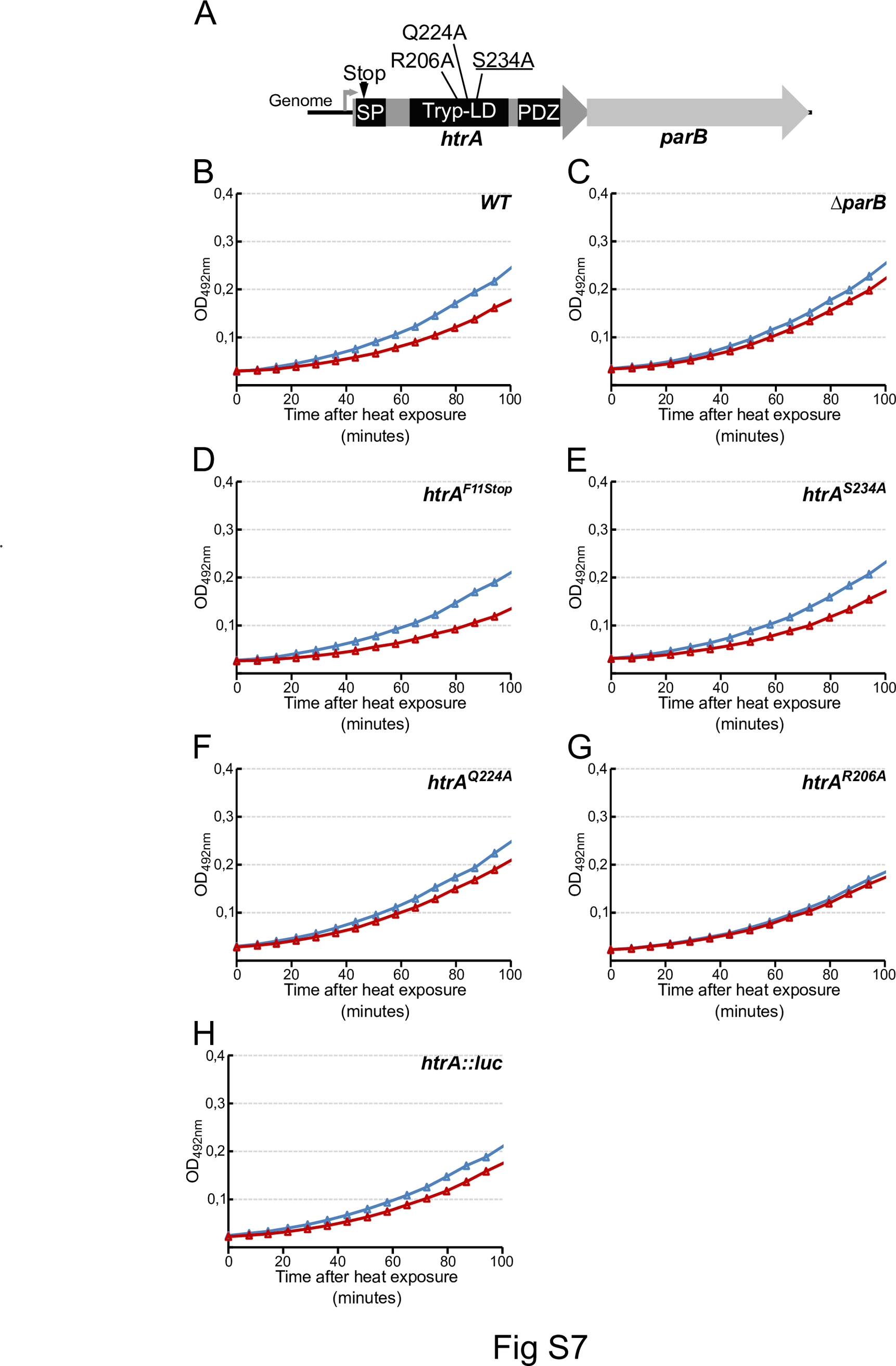

Supplement: S7 Fig — A. Schematic representation of the htrA/parB operon and position of relevant residues of HtrA. B-H. Growth of pneumococcal cells in Fig 7B–7H: B. R875 (comC::luc); C. R4657 (comC::luc, parB-); D. R4629 (comC::luc, htrAF11stop); E. R4630 (comC::luc, htrAS234A); F. R4676 (comC::luc, htrAQ224A); G. R4684 (comC::luc, htrAR206A); H. htrA expression in strain R2813 (htrA::luc). Red; 15 minutes exposure to 42°C. Blue; 15 minutes exposure to 37°C. (TIF) [file pgen.1010946.s007.tif]

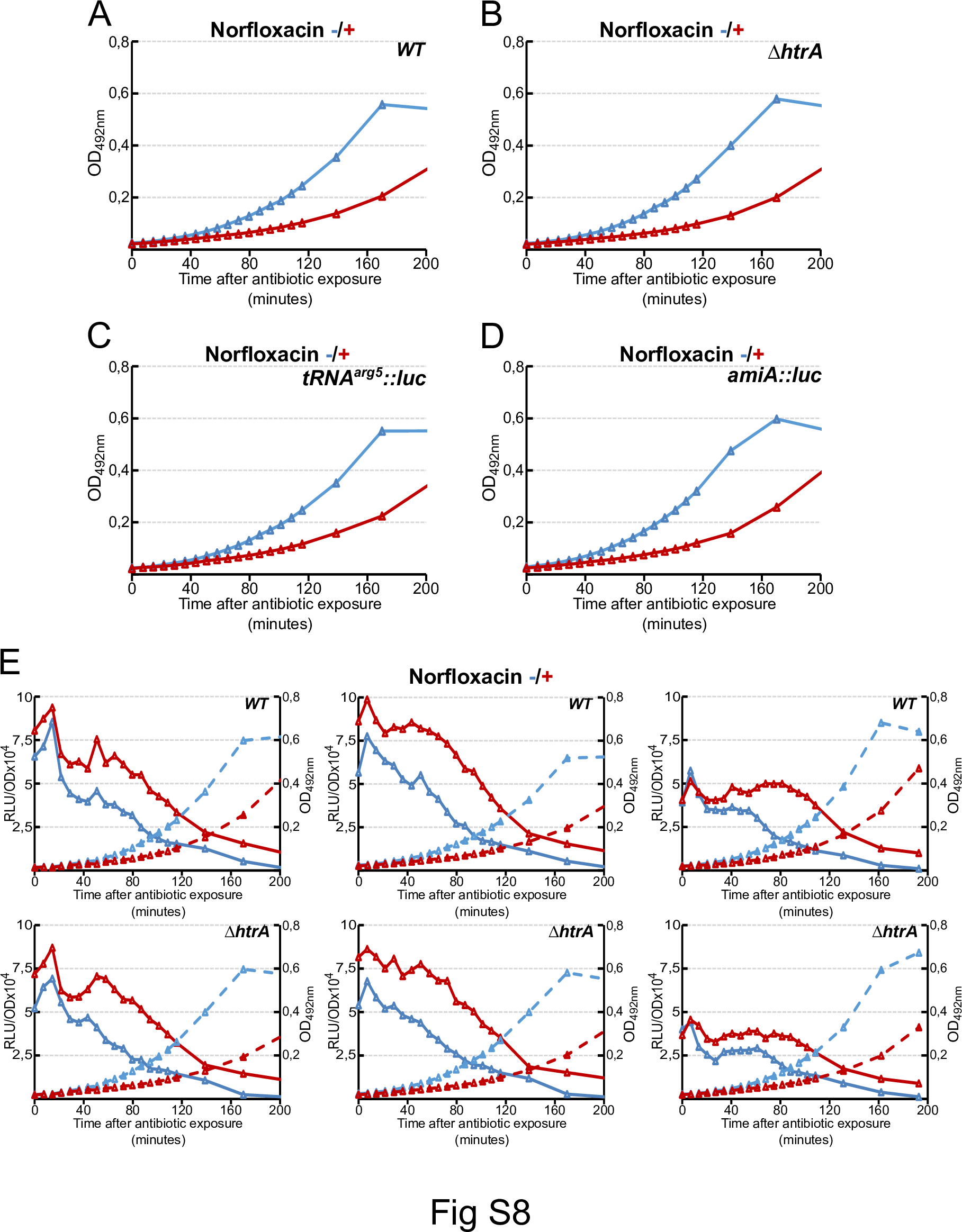

Supplement: S8 Fig — A-D. Growth of pneumococcal cells growing in C+Y medium at 37°C after 15 minutes exposure to Norfloxacin (1.25 μg mL-1) (red symbols) or no antibiotic (blue symbols) prior to first measurement. A. R875 (comC::luc); B. R4629 (comC::luc, htrA-); C. R4642 (tRNAarg5::luc) D. R4641 (amiA::luc). E. To illustrate reproducibility, the results from Fig 8 panel A and Fig 8 panel B are presented in the form of individual experiments carried out separately. comCDE expression shown as relative luminescence unit per OD (RLU/OD) in strains growing in C+Y medium at 37°C after 15 minutes exposure to Norfloxacin (1.25 μg mL-1) (red symbols) or no antibiotic (blue symbols) prior to first measurement (solid line). Dashed lines correspond to respective growth curves derived from OD492nm measurements. Upper panels, R875 (parental strain). Lower panels, R4629 (htrA-). (TIF) [file pgen.1010946.s008.tif]

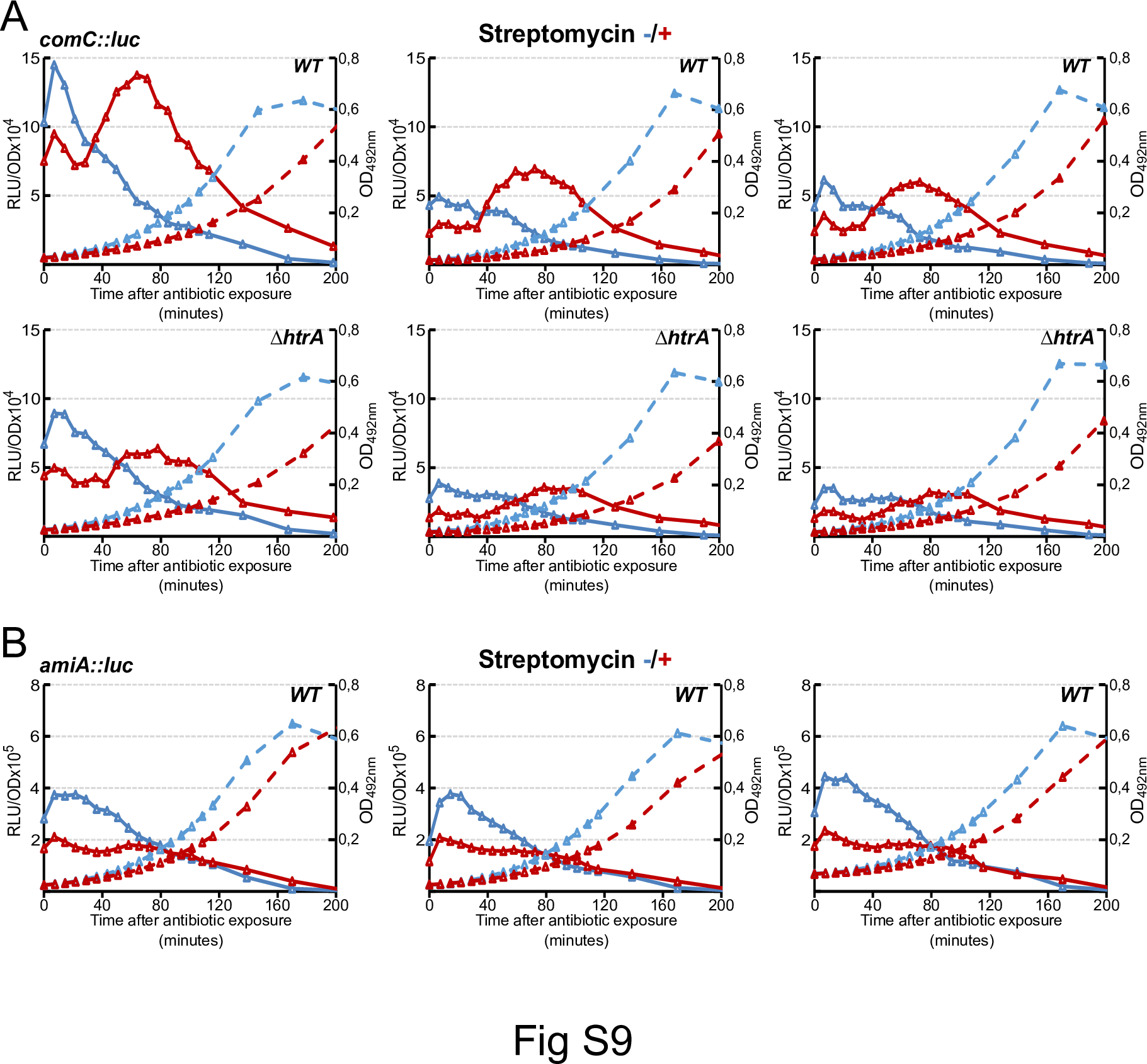

Supplement: S9 Fig — A. comCDE expression shown as relative luminescence unit per OD (RLU/OD) in strains growing in C+Y medium at 37°C after 15 minutes exposure to streptomycin (6.25 μg mL-1) (red symbols) or no antibiotic (blue symbols) prior to first measurement (solid line). Dashed lines correspond to respective growth curves derived from OD492nm measurements. Upper panels, R875 (parental strain). Lower panels, R4629 (htrA-). B. Same as A but for amiA expression (R4641). To illustrate reproducibility, the results are presented in the form of individual experiments carried out separately. Global fold induction (FI) for these three experiments is 1.55 +/-0.09 for strain R875, 1.03+/-0.05 for strain R4629 and 0.68+/-0.03 for strain R4641 (see materials and methods). (TIF) [file pgen.1010946.s009.tif]
