# Supplementary material for: Fever-like temperature bursts promote competence development via an HtrA-dependent pathway in Streptococcus pneumoniae
Source: PLoS Genet. 2023 Sep 12;19(9):e1010946. doi: 10.1371/journal.pgen.1010946 (PMC10516426; doi:10.1371/journal.pgen.1010946)
Supplement: S1 Table — (PDF) [file pgen.1010946.s010.pdf]

S1 Table: Strains.

Strain	Gentotype / relevant feature	References	Figures
R825	*comC::luc*	[1]	1a; 2a
R875	*comA::kan, comC::luc*	Derivated from strain R391 [3]	2a ; 8a
R895	*ssbB::luc*	[2]	1c
R1262	*comC::luc, htrA::spc19*	this study	6g
R1502	*comC0, ssbB::luc*	[4]	4f
R1521	*comC0, comC::luc*	[5]	3b; 4a; 5a; 6a; 7b
R1526	*comC0, comC::luc, upclpP::Spc93*	Derivated from strain R1054 [2]*	6c
R1548	*comC0, comA::luc*	This study	4b
R1627	*comC0, comC::luc, comE::spc20c*	[5]	5b
R1628	*comC0, comC:: luc, comE::spc20c, pmalM::comE*	[5]	5f
R1648	*comC0, comC::luc, ΔcomDE, pmalM::comE*	[5]	5e
R1694	*comC0, tRNA* ^{arg5} *::luc*	[5]	3c
R1798	*comC0, comC::luc, comE* ^{D58A} *, hexA::spc*	[5]	5d
R2017	*comC0, comC ::luc , dprA ::spc*	[4]	6b
R2200	*comC0, comX2::luc*	[6]	4c
R2218	*comC0, comX1::luc*	[6]	4d
R2448	*comC0, dprA::luc*	This study	4g
R2813	*comA::kan, htrA::luc*	This study	7i
R3688	*comC0, comW::luc*	[6]	4e
R4254	*comC0, ssbB::luc, p-comE-GFP*	[7]	2c, S6
R4578	*comC0 , comC::luc , htrA::spc19*	This study	6d
R4585	*comC0 , comC::luc, hexA::ery*	This study	5c
R4629	*comA::kan , comC::luc , htrA* ^{F11stop}	This study	7d; 8b
R4630	*comA::kan , comC::luc , htrA* ^{S234A}	This study	7e
R4639	*comC0 , amiA::luc*	This study	3c
R4641	*comA::kan, amiA::luc*	This study	8d
R4642	*comA::kan, tRNAarg5::luc*	This study	8c
R4657	*comA::kan , comC::luc , parB::spc*	This study	7c
R4676	*comA::kan , comC::luc , htrA* ^{Q224A}	This study	7f
R4684	*comA::kan , comC::luc , htrA* ^{R206A}	This study	7g
R5129	*comC0, ssbB::luc, p-comE-GFP, htrA::spc19*	This study	S6

* R1526 carries a transposon insertion in the promoter region of the *clpP* gene, resulting in virtually no ClpP production[2].

1. Bergé M, Moscoso M, Prudhomme M, Martin B, Claverys J-P. Uptake of transforming DNA in Gram-positive bacteria: a view from Streptococcus pneumoniae. Mol Microbiol. 2002;45: 411–421.
2. Chastanet A, Prudhomme M, Claverys JP, Msadek T. Regulation of Streptococcus pneumoniae clp genes and their role in competence development and stress survival. J Bacteriol. 2001;183: 7295–7307. doi:10.1128/JB.183.24.7295-7307.2001
3. Guiral S, Mitchell TJ, Martin B, Claverys J-P. Competence-programmed predation of noncompetent cells in the human pathogen Streptococcus pneumoniae: genetic requirements. Proc Natl Acad Sci U S A. 2005;102: 8710–8715. doi:10.1073/pnas.0500879102
4. Mirouze N, Bergé MA, Soulet A-L, Mortier-Barrière I, Quentin Y, Fichant G, et al. Direct involvement of DprA, the transformation-dedicated RecA loader, in the shut-off of

pneumococcal competence. Proc Natl Acad Sci U S A. 2013;110: E1035-1044. doi:10.1073/pnas.1219868110

5. Martin B, Granadel C, Campo N, Hénard V, Prudhomme M, Claverys J-P. Expression and maintenance of ComD-ComE, the two-component signal-transduction system that controls competence of Streptococcus pneumoniae. Mol Microbiol. 2010;75: 1513–1528. doi:10.1111/j.1365-2958.2010.07071.x
6. Martin B, Soulet A-L, Mirouze N, Prudhomme M, Mortier-Barrière I, Granadel C, et al. ComE/ComE~P interplay dictates activation or extinction status of pneumococcal X-state (competence). Mol Microbiol. 2013;87: 394–411. doi:10.1111/mmi.12104
7. Mortier-Barrière, I., Polard, P. & Campo, N. Direct Visualization of Horizontal Gene Transfer by Transformation in Live Pneumococcal Cells Using Microfluidics. Genes 11, E675 (2020).
