# Supplementary material for: Fever-like temperature bursts promote competence development via an HtrA-dependent pathway in Streptococcus pneumoniae
Source: PLoS Genet. 2023 Sep 12;19(9):e1010946. doi: 10.1371/journal.pgen.1010946 (PMC10516426; doi:10.1371/journal.pgen.1010946)
Supplement: S2 Table — (PDF) [file pgen.1010946.s011.pdf]

S2 Table: Oligonucleotides used in this study.

Oligonucleotide	Sequence	Use	See materials and methods
**Point mutation in HtrA (R206A and Q224A).**			
MBc409	AAGGTTAGGCGACCAAAACTGACAGAAAGA	*htrA* locus amplification	A1
MBc410	CCACAAGCTGTGAAAAATAATCCACAATGT	*htrA* locus amplification	B1
MB429	GTATCCAGTCTCAATGCTAATGTATCCTTAAAATC	R206A	A2
MB430	GATTTTAAGGATACATTAGCATTGAGACTGGATAC	R206A	B2
MB431	CTACAAAAGCCATCGCTACTGATACTGCTATTAAC	Q224A	A2
MB432	GTTAATAGCAGTATCAGTAGCGATGGCTTTTGTAG	Q224A	B2
**Point mutation in HtrA S234A and stop/frameshift.**			
MB411	CTTCTTCTATAATCCGTTGAAAGAAATAGTC	*htrA* locus amplification	A1
MB412	GCTATTAACCCAGGTAACGCTGGCGGCCCACTG	S234A	A2
MB413	CAGTGGGCCGCCAGCGTTACCTGGGTTAATAGC	S234A	B2
MB414	GATGATGACAATCCTAAACTTCCCCC	*htrA* locus amplification	B1
MM9	GAAACATCTAAAAACATAAATACAAAAAATGGTTTC	stop and frameshift in *htrA*	A2
MM10	GAAACCATTTTTTGTATTTATGTTTTTAGATGTTTC	stop and frameshift in *htrA*	B2
MB419	GCTACAAACTGTTCCAATTTAACTGTG	*htrA* locus amplification (with MB411)	B1
**Transcriptional fusions with the *luc* gene (restriction sites in bold).**			
MP146	GCCGCG**AAGCTT**GGCTCCATAATATCCATAGG	*comA::luc*	
MP147	AAGGCCT**GGATCC**CATGGCTAATGAAGCTACAC	*comA::luc*	
MP182	CCGCG**AAGCTT**TAACGAAATCACGGTCAGCAATTT	*htra::luc*	
MP183	GC**GGATCC**TGCCTCCATATGTTTGAATTACTGAAA	*htra::luc*	
dprA25	CTG**AAGCTT**TCCAACTGCAGAAAAGATTGTCAG	*dprA::luc*	
dprA26	ATA**GGATCC**ATAGTTTGTGATTTTCATAAATAAC	*dprA::luc*	
AmiAB1	ACTGCA**GGATCC**TGCAAGTACACCTGCTGC	*amiA::luc*	
AmiAHIII	ACTGCA**AAGCTT**GGTGTGAAAAGACATAG	*amiA::luc*	
***parB* and *htrA* gene inactivation by mariner mutagenesis.**			
ParB1	AATTTGAAATGATTTCTATCACAG	amplification for mariner *htrA/parB* mutagenesis	
ParB2	CTACTATATTCTTCTTGATTTGAA	amplification for mariner *htrA/parB* mutagenesis	
