# Supplementary material for: Fever-like temperature bursts promote competence development via an HtrA-dependent pathway in Streptococcus pneumoniae
Source: PLoS Genet. 2023 Sep 12;19(9):e1010946. doi: 10.1371/journal.pgen.1010946 (PMC10516426; doi:10.1371/journal.pgen.1010946)
Supplement: Attachment — Submitted filename: responses to reviewers- fever like bursts.pdf [file pgen.1010946.s012.pdf]

## Reviewer's Responses to Questions

A point-by-point response to each comment of the three reviewers is presented below in blue writing.

**Comments to the Authors:**
**Please note here if the review is uploaded as an attachment.**

**Reviewer #1**: In the manuscript titled, "Fever-like temperature bursts promote competence development via an HtrA-dependent pathway in Streptococcus pneumoniae", Maziero et al demonstrate that transient heat shock results in two effects on the competence pathway - an immediate CSP-independent, small increase in basal expression of early competence genes as well as a later, CSP-dependent induction of the entire competence pathway. The authors focus on the earlier, CSP-independent effect in their work. Previous work had shown that increases in comCDE basal expression were dependent on the ComDE TCS. In agreement with that work, authors show that heat-shock dependent increase in comCDE basal expression was indeed dependent on ComDE signaling. This increase in the basal expression of comCDE was HtrA-dependent. To test if this initial burst of ComCDE expression was a general stress response, authors tested if brief exposure to antibiotics (norfloxacin and streptomycin) also had a similar effect. While exposure with norfloxacin led to this small increase in comCDE basal expression, albeit in an HtrA-independent way; the effect of streptomycin was suspect. The authors investigate an interesting topic that is of high importance for the field. Their findings show potentially interesting results, however, major gaps remain in mechanistic understanding of the pathways that authors attempt to explore in this work (as below).

Major Comments –

1. The authors mostly focus on the immediate increase in basal expression of ComCDE that fails to result in an induction of the late competence genes (Fig 4). In the absence of this induction, it is unclear what is the physiological significance of this small induction in ComCDE expression.

In previous work (Martin et al., 2010), the authors have shown the existence of a basal level of the *comCDE* operon whose existence depended on the ComDE TCS. However, to our knowledge, no modulation by stress of the transcription level of this operon had been directly visualized until this work. In fact, modulation of this basal level was deduced following distant events. In this work, we make the link between stress, the increase in the basal level of transcription of the *comCDE* operon, the triggering of competence and natural transformation.

The physiological significance of the small and immediate induction of *comCDE* expression in response to HS is that it is necessary to the massive transcriptional induction of the early and late competence genes, which occurs lately during the growth.

Several described correlations support this interpretation,

Firstly, in Figure 1A. The higher the temperature, the greater the burst and, consequently, the earlier competence occurs (with the exception of 45°C, where growth is affected). This is also true for the exposure period (Figure 1B).

These later additional findings have been incorporated in the revised version of the manuscript and presented in Figure S1ABC and the text has been accordingly modified by adding the following sentence line 121: "To check that such HS competence triggers were biologically effective, we evaluated natural transformation under the different conditions, and found that only heat-exposed cells showed a significant rate of transformation (Figure S1ABC)".

How this HS-mediated coordination of competence mechanistically proceeds remains to be precisely established - as discussed in our reply to the following second major comment – except that we found that it strictly involves the HtrA protease.

In that sense, we added in Figure 6 a new result showing that in an *htrA*$^-$ mutant, no burst occurs after heat shock and consequently no great peak of *comCDE* transcription competence occurs (no detectable transformants).

2. Fig 1 – It remains unclear why basal increases in ComCDE levels observed immediately following heat shock, which results in increased ComC levels, not result in a positive feedback loop that activates the competence pathway? Is CSP not being secreted? Is it being degraded?

Understanding the temporal choreography of pneumococcal competence coordination in a growing cell population is clearly an important perspective to this work. This coordination is known to depend on the CSP.

Importantly, the HS-induced small burst of basal *comCDE* expression does not return back to the initial basal level of *comCDE* expression in a comC$^+$ prior its delayed massive induction, while it does so in a *com*C0 background. This further supports that the CSP is the limiting factor in the coordination mechanism of competence. (Figure 2)

To understand why the CSP-producing strain does not switch to the autocatalytic loop, we investigated whether it was the CSP that was limiting. A population was exposed 20 minutes after heat shock to a saturating amount of CSP (100ng/mL). The autocatalytic loop was immediately triggered, regardless of the strain. This suggests that CSP is indeed the limiting agent.

We added line143 "the addition of CSP 20 minutes after HS resulted in the immediate triggering of the autocatalytic loop, suggesting that the available CSP was indeed a limiting factor", and we showed these data in Figure 2S.

It is for this reason that, building on results previously published by Sebert's group showing that HtrA can degrade CSP *in vitro*, we propose in the discussion, a model in which HtrA is a key regulator of negative and positive factors that impact of development of competence.

Alternatively, or concomitantly, the CSP produced could also be stolen by other cells via cell-cell contact (Prudhomme et al. 2016).

This limitation of the CSP is undoubtedly biologically important, as it is one of the elements that enables the *S. pneumoniae* population not to switch instantly at the slightest stress or at the stochastic switch of a single cell towards competence. It therefore undoubtedly contributes to the population's ability to define a significant alert threshold.

The exact nature of this limitation remains to be determined, but we believe it goes beyond the scope of this work.

)3. It remains unclear how HtrA stimulates heat-shock induced increases in basal expression of comCDE. Many questions remain on this front –
a. Previous work (that authors cite in lines 283-288) has shown that HtrA negatively regulates competence development by degrading CSP. Since this work focuses on the CSP-independent increase in basal expression of ComCDE, it is unclear how HtrA is leading to the opposite phenotype – that of promoting competence development. Is it indeed the protease activity of HtrA that is responsible for this phenotype, and is so, what is the substrate that HtrA is acting on to result in this increase?

As HtrA/DegP degrades a wide range of protein substrates (Kim et al., Proc Natl Acad Sci U S A. 2012, Zhang et al., Biochem J. 2019 Dec), we propose that HtrA degrades an unknown inhibitor that "puts a brake" on ComDE activity. On the other hand, we do not reject the *in vitro* ability to HtrA to degrade CSP. First, we integrate in the discussion this capacity to explain why CSP seems to be produced in insufficient quantity to promote the autocalytic loop just after heat shock. Moreover, this protease activity that could alter the quantity of the available CSP may well add an additional regulation layer of competence induction by integrating further signals during growth that modulate populational triggering of competence as previously described by Sebert's group (Steven. 2011, Cassone. 2012).

For this reason, we have modified subtitle in Figure 8E by adding "CSP-independent pathways "

Fig 8E. Summary of characterized and potential molecular CSP-independent pathways driving *comCDE* basal expression.

b. How does HtrA impact the CSP-dependent induction of the entire competence pathway observed ~75 min post heat shock (phenotype described in Figs 1, 2B)? Is that also HtrA-dependent?

The answer is yes. To solve this question, we provide (Figure 6) new data that show that in an *htrA*$^-$ mutant in a *comC*$^+$ genetic background no burst occurs after heat shock and consequently no great peak of *comCDE* transcription competence occurs (no detectable transformants).

c. Lines 228-229: Authors state that HS could allosterically stimulate HtrA catalytic activity. However, to conclusively show that temperature regulates HtrA allosterically, reaction kinetics and Kd values will be needed.

We agree that biochemical analysis of these mutants is needed to rigorously state that these are HtrA mutants allosterically altered in their proteolytic activity. This is clearly beyond the scope of this manuscript as this is not what we want to state with these mutants.

We were simply wondering whether such mutants designed by homology with the highly related DegP protease would have the same negative impact on HS-mediated competence induction as

found with the HtrA catalytic point mutant. What we found is that they did so, reaching the aim pursued by this genetical analysis of competence regulation by HS.

In addition, we have modified the paragraph heading (line 307).

"Conserved allosteric activation residues of HtrA are necessary for transduction of the HS signal to competence induction."

4. Fig 8 and lines 237-243:
a. Why is the basal expression of ComCDE in the non-antibiotic treated samples (blue lines) about 1-2 log10-higher than the corresponding samples (37C) reported throughout the paper? Shouldn't these be comparable?

Yes, this is true, and we thank the referee for pointing at this difference. There are two things to be clarified.

First, the basal level of the corresponding samples (37C) is about $1.10^5$ RLU/mL. The basal expression of *comCDE* in the non-antibiotic treated samples (blue lines) is 10-fold lower. This was due to the difference in the protocol. The shift of temperature is a simple transfer from heat bath to another. On the other hand, in the case of antibiotic exposure, the antibiotic has to be removed by centrifugation and cell resuspension. Of course, the control undergoes the same treatment and this explains the drop in the basal level of *comCDE*.

Second, there was a graduation error on the streptomycin exposure graph: it should read RLU/ODx104. This has been corrected.

b. Data of a single replicate presented in Fig 7B shows high fluctuation in ComCDE expression upon streptomycin treatment. Without more replicates, it is difficult to assess whether brief streptomycin exposure indeed results in increased basal expression of ComCDE. Is the increase in expression statistically significant? Moreover, the effect of streptomycin-treatment the authors are alluding to in this figure materializes ~80 mins after antibiotic exposure, unlike the immediate effect observed post-heat shock. Due to lack of additional data, it seems plausible that this is a distinct mechanism at play.

We agree with this comment on the data presented in Fig 8B (not Fig 7B). Results in Figure 8 have also been questioned by reviewer 3. With the aim of answering all these questions we have modified the Figure 8.

The aim of figure 8 was to show that not all signaling pathways are HtrA-dependent. As the streptomycin experiments gave intermediate results, we have modified this figure.

We now focus only on norfloxacin exposure in Fig 8, for which the results are clearer. We added data showing that *tRNA* luc expression is also increased after HS, while the transcription of promotor of *ami*A gene, used as a control, remains unchanged. These results strongly suggest the conclusion that norfloxacin induces a burst of *comCDE* in a HtrA independent manner (as HtrA dependent pathway convey only a burst of PE promotor of *comCDE*).

Concerning streptomycin exposure, it is not fully surprising that the burst of transcription occurs later by comparison with HS.

First, in the case HS, the transition and the cell response from one temperature to the other might proceed very rapidly. By comparison, the elimination of the streptomycin, (which alters protein translation and therefore luciferase synthesis) from the cells by centrifugation will be not as effective.

Second, in this work, the interest of streptomycin being above all to show that a mixed response to stress in regard to competence induction and implication of HtrA can exist, we propose to move these data to an additional figure (Fig S8) and provide them in the form of biological triplicates, together with growth curves to shown that results are quite robust. These results might be interpreted by stating that a same stress could be convey *via* several pathways to induce competence. We figured such a mixed layers of streptomycin-mediated competence induction by question marks in Fig 8.

Minor Comments –

We modified all these minor comments in the revised version of the manuscript, as further explained in the following point by point answers.

1. Lines 192 and Fig 5A – Isn't the burst of transcription observed in a ComC- background as opposed to WT?

Yes, this has been accordingly modified in the text as follow: "a *comC*$^-$ background"

2. Mismatches in the strain numbers in the figure legends and the strain table. For instance, for Fig 5C, strain 4585 not listed in the strain table

We corrected it. We added R4585 in the strain table.

; fig 5E, Is R1648 ComD+ strain (as in legend) or ComE+ as in strain table. Please re-check throughout the manuscript.

This confusion is due to an inversion of the strains named in the Figure 5 legend. This has been corrected in the revised version of the manuscript.

In figure 5. Strains shown in Fig5A, Fig5C, Fig5E are parental strains of strains shown in Fig5B, Fig5D and Fig5F respectively.

Strains R4585 and R1798 are *hexA*$^-$ strains (invalidation of the mismatch repair system allows an easier construction of the ComED58A mutation as described in *Martin et al, Mol. Microbiology 2010* (reference 5 in table strains).

Strain R1648 and strain R1628. These strains were constructed to avoid any potential polar effect of Com deletion on ComE. (*Martin et al, Mol. Microbiology 2010*, reference 5 in table strains table)

In these strains, the *comE* gene is expressed ectopically (this is why the basal level of *comCDE* is about 10-fold lower). In addition, at the endogenous *comCDE* locus, the *comE* gene is inactivated (R1628). This strain is *comD*$^{+}$ and *comE*$^{+}$

In R1648 both *comDE* at the endogenous locus are deleted. This strain is *comD*$^{-}$ and *comE*$^{+}$ (thanks to the ectopic expression of ComE)

3. Lines 209-210 – Rephrase to… clpP mutants "do not abolish"…., since there seems to be a further increase in the ComCDE levels in the ClpP- strain.

This has been modified in the revised version of the manuscript as follow:

:", *dprA* and *clpP* mutations do not abolish the increased *comCDE* expression right after HS."

4. What is the difference between Figs 7B and 7H? The figure legend suggests it is the same strain. Further, I could not find a reference to Fig 7H in the text.

Fig7H was just a control of Fig7I (same experiment), but it is not necessary so we have removed it in the revised version of the manuscript.

5. Fig 8: Correct X-axis to time after "antibiotic" exposure.

This mistake has been modified in the revised version of the manuscript.

**Reviewer #2:** The manuscript by Maziero et al. reports a novel signaling pathway that allows Streptococcus pneumoniae to activate competence by sensing a temperature upshift (similar to fever). The authors show that this physical stress is perceived by the communication module ComCDE through activation of the chaperone/protease HtrA. This priming of ComCDE is than amplified later through the positive feedback loop via the pheromone CSP, resulting in the massive activation of the late competence phase. Nevertheless, the molecular mechanism (direct or indirect) ensuring the relay between HtrA activation and the ComCDE system remains to be elucidated. In addition, the authors show that this novel pathway of competence activation in the pneumococcus is not used by antibiotics targeting DNA replication and partially used by antibiotics targeting the ribosome. This work is of importance since the signaling pathways sensing physicochemical stresses resulting in competence development remain largely a black box in streptococci.
Globally, the manuscript is well-organized with convincing genetic experiments that demonstrate the relay between a temperature upshift, HtrA activation and ComCDE activation. The authors should carefully edit their manuscript for typographical errors (a non-exhaustive list is available in minor comments).
Major comments:
1/ The authors show that the late competence phase could be activated by a temperature upshift in pH conditions that do not lead to spontaneous transformation. However, I think that its important to show that transformation events are really taking place.

We agree and we provide data in Figure S1C showing that heat-shocked cells transform in contrast to non-heat-shocked cells.

In addition, it would be interesting to show that the transformation rate is responding to the increase in temperature or duration of the upshift.

We performed this experiment and found that it was indeed the case. We included these new results in Figure S1 A and B in the revised version of the manuscript.

We thank you for this suggestion because these results highlight the qualitative correlation between the small burst of *comCDE* expression, the intensity of competence development and transformation. This supports the biological significance of the burst of transcription of *comCDE*.

2/ Although the relay between activated HtrA and ComCDE could be complex, a simple model where activated HtrA directly interacts in the membrane with the histidine kinase ComD to modulate the phosphorylation level of ComE could be easily tested either using a bacterial two-hybrid system or Split-nanoluc (functional in the pneumococcus). Although not fully required, such experiments could reinforce the story and give additional novelty.

We agree that ComD could be a good candidate, but it remains a candidate among others.

We are planning to carry out this type of experiment, which would provide good evidence for launching a project aimed at identifying this inhibition effect. However, as reviewer 2 says, we feel that this work is beyond the scope of this publication.

Minor comments:
L18-L19: “in the physical conditions of the environment, oxidative potential and temperature”. I do not see the point here to cite oxidative potential. I would remove “oxidative potential and temperature”. Change in “in physical conditions of environment”

Modified as reviewer requirement

L22: “We find that certain other stimuli”. This is unprecise and needs to be rephrase.

We agree, we modified as follows

“We find that other competence induction stimuli, such as that induced by norfloxacin, are not conveyed by the HtrA-dependent pathway”

L24: “by any of several independent pathways”. Again, lack of precision

We agree and we have modified accordingly as follow:

“This finding strongly supports that competence can be induced by at least two independent pathways

L39: “is essential to relaying of the thermal signal” -> is essential for relaying the thermal signal

This has been modified

L84-85: “such has that of Lee” -> such as. “That proteins” -> those proteins

This has been modified

L85-86: rephrase the sentence -> “, suggesting cross regulation

This has been modified as follow:

“The fact that those proteins are known to be involved in temperature adaptation, e.g. HtrA and HrcA, belong to the CiaRH and competence regulons (2,23,24) suggests cross regulation between stress-response pathways.”

L108: give the initial pH value here

This has been modified, i.e. pH 7.0

L142: “with a comC-minus strain” -> the minus superscript is not clearly visible

This has been modified. We doubled the spacing before the minus superscript

L190: “luciferase” -> remove italics

This has been modified

L224: “two key residues” -> cite those two residues here

These have been cited as follow: “are conserved in *S. pneumoniae* HtrA (R206 and Q224)”

L343: remove “, this”

This has been modified

L398-399: remove “was calculated as follows”

This has been modified

L424: performed with

This has been modified

L625-628: Fig 2A and B. Please indicate how many independent repetitions were performed

We mentioned in the legend of the figure that the experiment has been performed in triplicate as follow:

"For clarity, only a single data set, representative of three independent determinations made on different days, is presented".

**Reviewer #3:** In this manuscript the authors study the role of thermal stress on competence development in the pneumococcus. To do this, they track the expression of several competence genes after a short exposure to high temperature and find that heat shock induces a burst in expression of the early competence genes that is mediated by ComDE. Using a set of deletion mutants they explore which other genes might mediate this induction and identify htrA as a potential candidate for transmitting the heat shock signal to the competence operons. Although previous studies had already established a link between temperature and competence regulation (e.g. Luo et al 2003),

We added this reference in the introduction (line 89).

this manuscript systematically quantifies this effect across different competence genes and sheds light on a potential mechanism by identifying that induction of the com genes occurs through the PE promoter. While the evidence that they present in this respect is convincing, I find that the data on the involvement of htrA is less solid. In fact, I was surprised by their findings regarding HtrA given that previous work has shown that this protein degrades CSP and potentially ComD and thus might inhibit rather than induce competence development. The main source of evidence for the presence of an HtrA-dependent pathway is that the initial burst in comCDE expression is not observed in htrA deletion mutants after heat shock. However, since the authors do not present the OD data it is unclear whether the htrA deletion mutants have a growth defect during the first 100min (which in fact wouldn't be surprising given that previous studies have identified such growth defects upon thermal stress when htrA is deleted).

We agree and provide all growth curves in the supplementary figures. Transient exposure to heat does not significantly affect the growth of the *htrA* mutant compared to the parental strain (e.g., Figure 6).

In addition, the use of RLU/OD makes it possible to integrate growth variations between different strains or mutants by reflecting a real specific activity per cell.

Moreover, this experiment was done using a comC deletion background which prevents the appearance of the second large competence peak that the authors observe when the wildtype is exposed to thermal stress (Figure 1A). In this sense, I could imagine that an htrA deletion mutant in a WT background upon exposure to 42C could look like the WT exposed at 45C

(Fig 1A). In this case, the initial burst of comC expression is not observed because of the long lag but the second competence peak appears once the culture grows. The authors should construct the htrA deletion mutant in a WT background and show that competence is never induced (as it happens when the cultures are not exposed to heat) to confidently claim that HtrA is necessary for the heat shock signal to be transmitted to the com operon.

We agree, and we provide Data in Figure 6 that demonstrate that an *htrA*$^{-}$ strain (in a *comC*$^{+}$ genetic background) is unable to trigger competence even after heat exposure. We also checked that no transformation (under the detection level) occurs in this strain.

Moreover, no significant growth defect could be detected in an *htrA*$^{-}$ mutant compare to its parental strain.

Moreover, to further disentangle the effect of deleting htrA on growth upon heat stress and competence induction I'd suggest that the authors perform the single cell competence assays done in Fig 2 with the htrA deletion mutants and show that the fluorescence distributions are similar in heat exposed and unexposed cultures.

We performed and added this experiment in Figure S6. In the *htrA* mutant, heat shock does not promote the increase in GFP fluorescence as it does in the wild-type isogenic parental strain. This confirms our previous observations with the luciferase reporter.

In order to compare the behavior of the *htrA* and wild-type strains as accurately as possible, we performed these experiments in parallel. This led us to replace the wild-type data set in figure 2 with these new data. This has no impact on interpretation.

Also, the fact that htrA can 'transmit' antibiotic stress upon exposure to Streptomycin but not Norfloxacin could be constant with the explanation above: HtrA might be necessary to prevent growth defects upon exposure to Streptomycin given that this antibiotic interferes with protein synthesis unlike Norfloxacin which targets DNA replication. Thus, deleting htrA might affect growth upon exposure to Streptomycin which can in turn translate in the absence of an initial burst of competence induction not because HtrA is directly interfering and transmitting signals to the comCDE operon but rather because of the general physiological state of the cells.

Results presented in Figure 8 was also questioned by reviewer #1. To answer these similar concerns we propose to modify this figure, as explained in following.

The aim of figure 8 was to highlight that not all signaling pathways are HtrA-dependent. As the streptomycin experiments gave intermediary results, we propose to modify this figure.

We now focus only on norfloxacin exposure in Fig 8, for which the results are clearer. We added data that show *tRNA* luc expression is also increased after HS, while the transcription of promotor of *amiA* gene, used as a control, remains unchanged. This results strongly suggest the conclusion that norfloxacin induces a burst of *comCDE* in an HtrA-independent manner (as HtrA-dependent pathway convey only a burst of PE promotor of *comCDE*).

Concerning streptomycin exposure, it is not fully surprising that the burst of transcription occurs later by comparison with HS. First, in the case HS, the transition and the cell response from one temperature to the other might proceed very rapidly. By comparison, the elimination of the streptomycin, (which alters protein translation and therefore luciferase synthesis) from the cells by centrifugation will be not as effective.

Second, in this work, the interest of streptomycin being above all to show that a mixed response to stress in regard to competence induction and implication of HtrA can exist, we propose to move these data to an additional figure (Fig S8) and provide them in the form of biological triplicates, together with growth curves to shown that results are quite robust. These results might be interpreted by stating that a same stress could be convey via several pathways to induce competence. We figured such a mixed layers of streptomycin-mediated competence induction by question marks in Fig. 8.

These experiments were
performed in the comA deletion background so as before I'd suggest
that the authors repeat this with htrA deletion mutants in a WT
background and assess competence development through the full growth
curve.

Streptomycin has already been shown to promote the development of competence a wild-type genetic context (Prudhomme et al. 2006), and CSP has been reported to be implicated in this phenomenon (Stevens 2011).

Given the complexity of streptomycin's biological effects, characterizing the different pathways inducing competence would require a comprehensive study that would not be solved with a single experiment.

Our aim was to test whether HtrA is implicated in streptomycin-mediated competence induction.

We found that it was partially the case, highlighting that multiple signaling pathways could be involved in response to a given competence-inducing stress. We figured such a mixed layers of streptomycin-mediated competence induction by question marks in Fig 8. Also, we have moved these data to an additional figure (Fig S8) to provide them in the form of biological triplicates, together with growth curves, which show that results are quite robust

We modified the legend in Fig 8 by adding "CSP-independent pathways" in the subtitle of Fig 8E, to highlight that this work focusses on CSP-independent pathway that drive *comCDE* basal expression:

Fig 8C. Summary of characterized and potential molecular CSP-independent pathways driving *comCDE* basal expression.

Other comments:

-Line 151. Unclear phrasing.

We have modified this sentence as follow:

Quantification of mean fluorescence per cell further revealed a significant increase of GFP synthesis upon HS in all cells. This higher level of GFP remains heterogeneous as observed before the HS (Figs 2DE).

- Line 156. To streamline the story I'd suggest that the data showing that the PE promoter is involved in competence induction by heat shock and the data showing that ComDE is necessary for the upregulation of competence genes upon heat stress (now in Figures 3 and 5) are presented together.

This was our initial intention, but after several attempts, we found that it generated a too large paragraph, disrupting the equilibrium of the manuscript. Also, we prefer the current presentation.

- The accompanying growth curves should be presented for all figures either in the SI or in the main text (the authors could add another axis to RLU/OD plots to have both pieces of the data in the same figure instead of creating an additional panel)

We have included all the growth curves in supplemental figures in the revised version of the manuscript.

- Use a log scale for the RLU/OD values. In this way you can also use the same axis range for all plots which would be especially useful for comparing panels in the same figure (e.g. Fig 2).

We have attempted this representation, but it graphically overwrites the initial burst of early gene transcription. The enlargement we propose in Fig 2B is intended not only to give a better view of the burst of *comCDE* expression, but also to introduce the new format of subsequent figures (from figure 3 to the end). For these reasons, we prefer the current presentation.

- Use consistent notation for gene deletions (e.g. Fig 6)

In Figure 6, the clpP mutant was indicated as *clpP⁻* and not Δ*clpP*, because a Δ*clpP* strain does not growth in the C+Y medium used to allow competence development (Chastanet et al., J. Bacteriol 2001). We used a strain that contains an insertion in the promoter region of *clpP* that provokes a large decrease of ClpP production (Chastanet et al., J. Bacteriol 2001). We propose to keep this annotation and add this additional information in the strains table.

- Figure 7. What is the difference between panels A and H? and if they're different what's the purpose of the data presented in H?

Fig7H was just a control of Fig7I (same experiment). As it appears not necessary, we have removed it in the revised version of the manuscript.

- Figure 8. Legend should read 'Time after antibiotic exposure'. 'Time after antibiotic exposure'. This has been corrected in the revised version of the manuscript

Also,
shouldn't the blue curves in panel A and B be the same in panels A and B? There is a 10-fold difference in the RLU/OD signal and as far as I understand these correspond to either the WT or the htrA deletion mutant in the absence on antibiotic exposure and thus should be the same conditions in panels A and B.

Thanks for this comment. This was due to a graduation mistake on the streptomycin exposure graph: it should read RLU/ODx$10^4$. This has been corrected in the revised version of the manuscript.

- Line 399. Is this done with the RLU or RLU/OD values? Please give more details on how the calculation is done.

We rephrased this description as follow.

"Where appropriate, fold induction (FI) of the expression of the various genes of interest was calculated as the ratio of the area under the curve of heat-exposed cells compared to the area under the curve of unexposed cells. The areas under the curve were estimated by summing the values of the luciferase activity values (RLU/OD) over the first 60 minutes of the experiment. The means of the ratio and standard deviation are the averages of at least three independent determinations made on different days."

---

**Have all data underlying the figures and results presented in the manuscript been provided?**
Large-scale datasets should be made available via a public repository as described in the *PLOS Genetics* data availability policy, and numerical data that underlies graphs or summary statistics should be provided in spreadsheet form as supporting information.

Reviewer #1: **No:** Authors only show data for a single replicate throughout the manuscript.

Reviewer #2: Yes

Reviewer #3: **No:**

---

PLOS authors have the option to publish the peer review history of their article (what does this mean?). If published, this will include your full peer review and any attached files.

If you choose "no", your identity will remain anonymous but your review may still be made public.

**Do you want your identity to be public for this peer review?** For information about this choice, including consent withdrawal, please see our Privacy Policy.

Reviewer #1: No

Reviewer #2: No

Reviewer #3: No
