# Supplementary material for: Fever-like temperature bursts promote competence development via an HtrA-dependent pathway in Streptococcus pneumoniae
Source: PLoS Genet. 2023 Sep 12;19(9):e1010946. doi: 10.1371/journal.pgen.1010946 (PMC10516426; doi:10.1371/journal.pgen.1010946)
Supplement: Attachment — Submitted filename: response to reviewer.pdf [file pgen.1010946.s013.pdf]

Reviewer #1: The authors have addressed my concerns.

Reviewer #3: I thank the authors for providing the additional growth data and performing the single-cell experiments in the htrA mutants. Some additional comments below:

1. After re-reading the abstract, I think the authors should remove the claim that 'there have been no reports of the response of the pneumococcal competence regulon to changes in the physical conditions of the environment'. The effect of environmental variables such as pH and environmental diffusivity has been thoroughly explored before (Yang et al 2010; Domenech et al 2020; Moreno-Gamez et al 2017) and as I mentioned in my previous review even the role of temperature is not something that the authors are establishing for the first time.

We agree and we have withdrawn this sentence from the abstract.

Concerning temperature, we have modified the introduction (line 87) to clarify this point.

"This observation and other work (25–27) raise the possibility of a link between temperature and competence".

2. I found it surprising that the htrA mutant does not only lack a growth defect upon heat stress but it even seems to grow better than the WT (fig 6F vs 6H). I'd appreciate if the authors include a sentence in the discussion about this. I did a quick search and couldn't find studies reporting similar observations upon deleting htrA - deletion mutants always seem to underperform under stressful conditions relative to the WT.

It's true that most of the time, under stress conditions, an *htrA* mutant will show more or less impaired growth. However, it should be noted that this can vary greatly depending on the composition of the medium. We also noticed a growth delay in *htrA* in prolonged growth at 39°C or 40°C in C+Y medium

.

Indeed, the most important point to note is that after ***transient heat stress***, cells were cultivated under "non-stressing" conditions (37°C). In these conditions, we didn't observe any significant growth delay for the *htrA*$^{-}$ mutant in comparison with the wildtype strain. In addition, the transient temperature stress we mostly used in our study prior to growth at 37°C , i.e 15 min at 42°C, is well known to block the growth of wild-type cells when applied continuously (Charpentier *et al*., 2002), a result that we reproduced with our strains including the *htrA* mutant. Also, when the strains were cultured at 37°C, we did not notice any growth defect.

It even seems, as you note, that the heat-shocked wild-type strain grows slightly slower than the *htrA* strain, but this is because the heat-shocked wild-type strain has become competent, whereas the *htrA* strain has not. It's well established that becoming competent leads to transient growth delay (Ephrussi-Taylor H., 1964, Havarstein et al., 2006, Mirouze et al., 2013, Bergé et al, 2017).

In all, it seems that under these transient stress conditions, HtrA does not play a major role in growth recovery.

Such approach of transient stress exposure offers the advantage of being able to follow the onset of competence while limiting the impact of this stress on growth.

In conclusion, we propose to include this remark in the legend of figure 6.

"Note that the *htrA*⁻ strain does not show significant growth retardation, as growth takes place at 37°C following transient exposure at high temperature."

3. Regarding my suggestion about using a log scale I don't understand what the authors mean with '... it graphically overwrites the initial burst of early gene transcription.', especially since the log-scale would have precisely the opposite effect of amplifying that initial burst (e.g. in Figure 2A the two-fold increase in expression level that the authors observe would be ~1/4th of the second peak).

We agree that we weren't clear enough in our answer. We wanted to emphasize that a logarithmic representation over 300 minutes obscures the two luciferase expression peaks due to the low RLU values at the end of growth, (as seen in the following figure, in the lower left panel). This problem can be solved by limiting the period studied, but this would be awkward for Figure 1, in which the peaks do not systematically occur at the same time. Alternatively, the lowest values can also be eliminated (as seen in the following figure, in the lower right panel).

Then the two peaks recorded during the growth appear to be of the same size. We think this is graphically misleading. It's important for us to point out that these two "peaks" are not equivalent. The first is an increase in the base level of comCDE expression, while the second is the massive and "true" peak of competence, classically recorded via this approach, corresponding to the synchronization of cells by the CSP.

This is why we prefer the linear representation (upper panel). In addition, the creation of "panel B" introduces the representation that will be used systematically from figure 3 onwards.

Of course, if this still remains determinant crucial issue, the logarithmic form could be used.

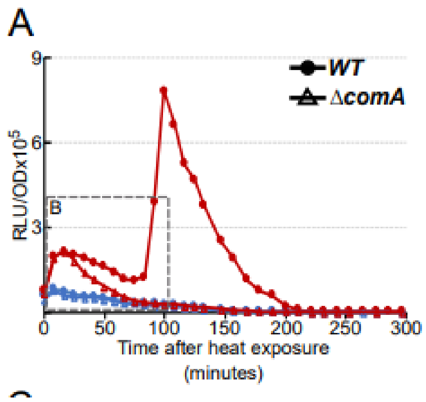

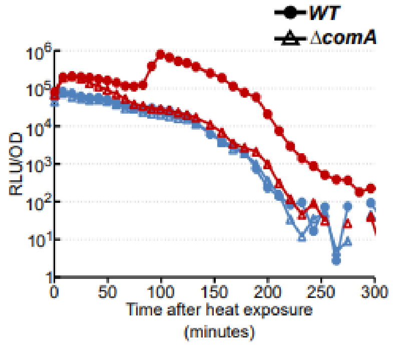

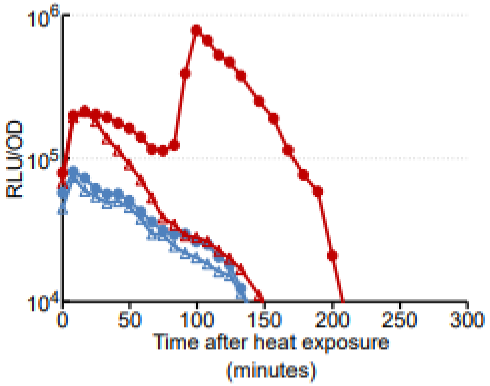

3b- Also, as I suggested the first time please use the same y-axis range in all panels in the same figure because otherwise it becomes hard to compare them.

We agree that Y-axis standardization is more correct when the transcription reporter is the same (e.g. *comC*::*luc*, Figure 5 and Figure 7, Figure S8 now Figure S9). We have modified it as required.

But as the reporter fusions are not the same in other figures, the basal level is not the same (e.g. *comC*::*luc*, *comX*::*luc*, *comW*::*luc*, etc.)

Also, as observable variations are more qualitative (presence or absence of a burst) than quantitative (intensity of the burst), we have chosen to favor a graphical representation adapting the Y axis according to the base level expression. The advantage of such a representation is that it avoids masking potential bursts of base level lower than that of *comC*::*luc* (Figure 3, Figure 4 and Figure 6)

4. Given the variability observed for Streptomycin it'd be great if the authors show additional replicates for the Norfloxacin data. It seems like now the authors are presenting data from only a single experiment.

This is right, and as such we have now added in Figure S8E the individual experiment to support the reproducibility of the effect of norfloxacin on *comCDE* transcription and the non-requirement of HtrA in this phenomenon.

5. Line 303. It'd be great if the authors could reflect on why their results are different from the ones obtained by Stevens et al besides just stating the observations are not consistent among both studies.

We did not compare the results obtained by Stevens et al because we felt that our study was too different from their's, explaining why we had limited ourselves to factual observations. However, we understand the confusion this may cause.

Many points could explain the variation in *htrA* phenotype observed between these two studies.

First, we didn't study the same causal relation between stress and competence induction. We analyzed how a transient heat stress impacts the temporality of competence development and Stevens et al. focused their analysis on how a continuous alteration of protein biosynthesis accuracy during growth leads to competence induction.

Accordingly, we propose that HtrA has a double impact on competence development; a positive one through up-regulation of *comCDE* transcription (our present work) and a negative one through its ability to degrade secreted CSP (Cassone et al,.).

We therefore propose that, depending on the stimulus tested and the culture conditions, including the continual or transient maintenance of the competence inducing stress during the growth, one of these two ways of the regulation driven by HtrA would dominate over the other. In the case of a transient heat stress, we propose that up regulation of *comCDE* is the predominant mechanism. In contrast, in the case of a translation fidelity stress continuously maintained during the growth, it seems HtrA-directed CSP degradation predominates (Steven et *al.*,).

To clarify this point we added line 349, after the presentation of our model,

"HtrA would therefore have two actions on competence induction, one positive through its ability to increase *comCDE* transcription, and the other negative through CSP degradation. Depending on the stimulus and physiological conditions analysed, one of these actions would predominate. Thus, in the case of modulation of translation accuracy (41), degradation of CSP would be the dominant element regulating the onset of competence."

6. Line 354. Looking at figure S8 I'm not sure to which initial burst the authors are referring for the Streptomycin data. In fact, it looks like that first burst is reduced in the Streptomycin treatment relative to the control and only later the full peak appears. Please clarify this.

Just after streptomycin treatment, we noted a decrease in the basal level of transcription of the *comCDE* operon in the streptomycin-treated sample, but this is not specific to the *comCDE* operon since the same effect can be observed on control genes such as *amiA::luc* (we have added this data in a new Fig S9). This phenomenon may be attributed to the antibiotic activity of streptomycin, in particular its anti-translational activity, which must affect the synthesis of the luciferase reporter. A certain amount of time is then required for the treated sample to return to a normal transcription level, as observed with the *amiA::luc* gene (approximately 50 to 80 minutes; Fig. S9).

Nonetheless, during this initial period following streptomycin treatment, a significant increase in the transcription of the *comCDE* operon was observed. This increase is independent of CSP (as this strain could not produce CSP) and therefore cannot be the "full peak" of competence (Figure 1 and Figure 2), which results from the synchronization of the whole population under the action of CSP.

It is therefore reasonable to propose that streptomycin induces a specific increase of the basal level of *comCDE* transcription. Admittedly, this phenomenon is delayed compared to what has been observed following transient exposure to heat or norfloxacin, but this seems most probably due to the transient anti-translational action of streptomycin.

To clarify this point, we have added data from a strain carrying the *PamiA::luc* control gene in a new Figure S9. This figure groups all the data concerning exposure to streptomycin.

7. There is a typo in the y-axis labels of Figure S8.

This error has been corrected. We have replaced DO with OD in this figure (now Figure S9)
